# Halogen Bond-Assisted Supramolecular Dimerization of Pyridinium-Fused 1,2,4-Selenadiazoles via Four-Center Se_2_N_2_ Chalcogen Bonding

**DOI:** 10.3390/ijms25073972

**Published:** 2024-04-03

**Authors:** Evgeny A. Dukhnovsky, Alexander S. Novikov, Alexey S. Kubasov, Alexander V. Borisov, Nkumbu Donovan Sikaona, Anatoly A. Kirichuk, Victor N. Khrustalev, Andreii S. Kritchenkov, Alexander G. Tskhovrebov

**Affiliations:** 1 Research Institute of Chemistry, Peoples’ Friendship University of Russia, 6 Miklukho-Maklaya Street, Moscow 117198, Russia; 2 Institute of Chemistry, Saint Petersburg State University, Universitetskaya Nab. 7/9, Saint Petersburg 199034, Russia; 3 Kurnakov Institute of General and Inorganic Chemistry, Russian Academy of Sciences, Leninsky Prosp. 31, Moscow 119334, Russia; 4 Department of Chemistry, R.E. Alekseev Nizhny Novgorod State Technical University, Minin St., 24, Nizhny Novgorod 603155, Russia; 5 N.D. Zelinsky Institute of Organic Chemistry, Russian Academy of Sciences, Leninsky Prosp. 47, Moscow 119334, Russia

**Keywords:** selenadiazoles, non-covalent interactions, hydrogen bonding, halogen bonding, chalcogen bonding

## Abstract

The synthesis and structural characterization of α-haloalkyl-substituted pyridinium-fused 1,2,4-selenadiazoles with various counterions is reported herein, demonstrating a strategy for directed supramolecular dimerization in the solid state. The compounds were obtained through a recently discovered 1,3-dipolar cycloaddition reaction between nitriles and bifunctional 2-pyridylselenyl reagents, and their structures were confirmed by the X-ray crystallography. α-Haloalkyl-substituted pyridinium-fused 1,2,4-selenadiazoles exclusively formed supramolecular dimers via four-center Se···N chalcogen bonding, supported by additional halogen bonding involving α-haloalkyl substituents. The introduction of halogens at the α-position of the substituent R in the selenadiazole core proved effective in promoting supramolecular dimerization, which was unaffected by variation of counterions. Additionally, the impact of cocrystallization with a classical halogen bond donor C_6_F_3_I_3_ on the supramolecular assembly was investigated. Non-covalent interactions were studied using density functional theory calculations and topological analysis of the electron density distribution, which indicated that all ChB, XB and HB interactions are purely non-covalent and attractive in nature. This study underscores the potential of halogen and chalcogen bonding in directing the self-assembly of functional supramolecular materials employing 1,2,4-selenadiazoles derived from recently discovered cycloaddition between nitriles and bifunctional 2-pyridylselenyl reagents.

## 1. Introduction

The creation of functional supramolecular materials with programmable structures and, as a result, tunable properties through a bottom-up approach has posed a persistent and enduring challenge. Among the numerous supramolecular linkages employed for creating complex assemblies, coordination and hydrogen bonds are the most extensively utilized, which resulted in the rise of metal–organic frameworks (MOFs) [1,2] and hydrogen-bonded organic frameworks (HOFs) [3,4,5,6,7]. In recent years, halogen bonding (XB) and chalcogen bonding (ChB) have emerged as potent alternatives for hydrogen bonding, due to their directionality and superior tunability [8,9,10,11,12,13,14,15,16,17,18,19,20,21,22,23,24,25,26,27,28,29,30]. Despite their potential benefits, XB and ChB have garnered significantly less attention in the context of the creation of extended materials akin to HOFs and MOFs [30].

Chalcogenodiazoles are appealing candidates for creating such materials [31,32,33,34,35,36,37]. They have demonstrated the ability to assemble into symmetrical antiparallel supramolecular dimers through two Ch···N chalcogen bonding interactions. These appealing supramolecular building blocks have undergone extensive investigation in recent years [32,33,34,35,36,37].

Recently we have described a novel 1,3-dipolar cycloaddition reaction between nitriles and bifunctional 2-pyridylselenyl reagents, which allows us to synthesize otherwise inaccessible pyridinium-fused 1,2,4-selenadiazoles [38,39,40]. The latter showed a propensity to self-assemble into antiparallel supramolecular dimers in the solid state via four-center Se_2_N_2_ ChB. The formation of dimers was not observed for all the structurally characterized cationic selenadiazoles and depended on the substituents in the heterocyclic system [41,42,43,44]. In some cases, the square formation was outcompeted by other weak intermolecular contacts in the solid state. This prompted us to search for approaches for directed supramolecular synthesis involving our novel synthons featuring four-center Se_2_N_2_ ChB.

In a previous work [43], we demonstrated that benzylic-substituted pyridinium-fused 1,2,4-selenadiazoles exclusively form supramolecular dimers via four-center Se_2_N_2_ and two symmetrically equivalent selenium···arene ChB interactions. This benzylic substitution approach can be employed for reliable supramolecular dimerization of pyridinium-fused selenadiazoles in the crystal, which can be applied in supramolecular engineering.

Here, we report the synthesis and structural characterization of α-haloalkyl-substituted pyridinium-fused 1,2,4-selenadiazoles with various counterions and demonstrate that the introduction of a halogen at the α-position of substituent R in the selenadiazole core may be an effective strategy for directed supramolecular dimerization of selenadiazoles in the solid state.

## 2. Results and Discussion

**Scheme 1:**
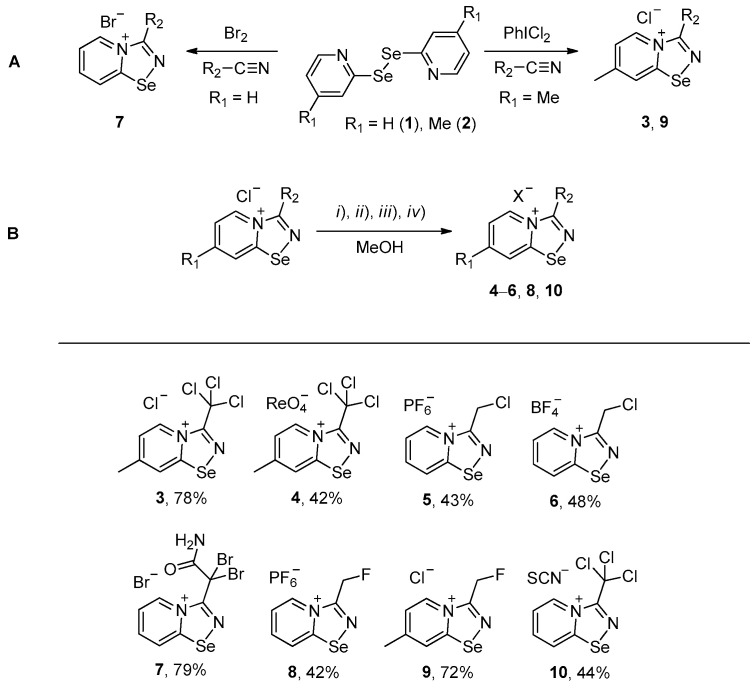
Synthesis of **3**–**10**. (i) HReO_4_, (ii) NBu_4_PF_6_, (iii) HBF_4_, (iv) NH_4_SCN.

Halides of *α*-haloalkyl-substituted pyridinium-fused 1,2,4-selenadiazoles were obtained by the oxidation of 2,2′-dipyridyl diselenide **1** or 4,4′-dimethyl-2,2′-dipyridyl diselenide **2** followed by sequential cyclization of in situ generated 2-pyridylselenyl halide or 4-methyl-2-pyridylselenyl halide with corresponding *α*-haloalkylnitriles (Scheme 1A, see experimental part for details). The salts of ReO_4_^−^, PF_6_^−^, BF_4_^−^ and SCN^−^ were obtained via anion metathesis in 1,2,4-selenadiazolium chlorides (Scheme 1B).

**Figure 1:**
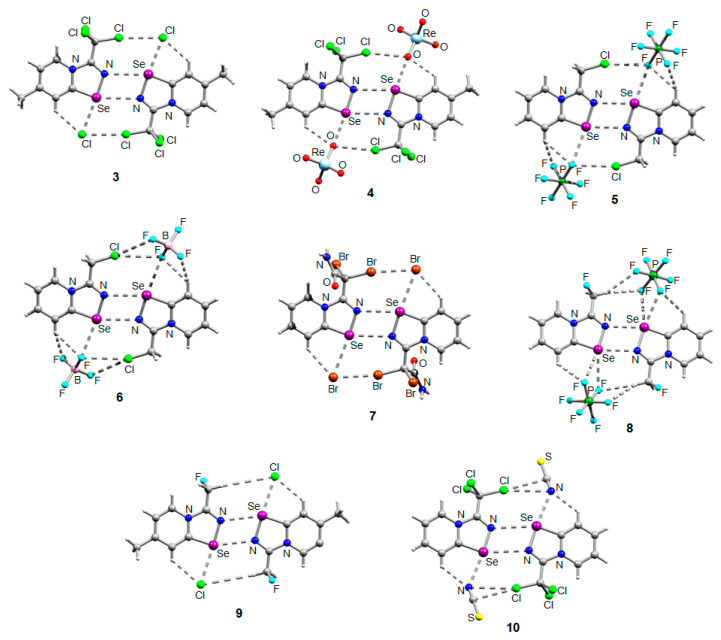
Ball-and-stick representations of the crystal structures of **3**–**10** demonstrating supramolecular dimerization via Se···N ChB assisted by the XB or HB (for **8** and **9**). Grey and light-grey spheres represent carbon and hydrogen, respectively.

The NMR data for **3**–**10** was consistent with the proposed structures. Compounds **3**–**10** could be recrystallized from the MeOH-Et_2_O mixture to give single crystals suitable for X-ray structural analysis, which confirmed their structures (Figure 1).

The crystal quality for **6** did not allow us to establish precise metrical parameters, but confirmed the atom connectivity in the solid state. Structural analysis revealed that for **3**–**10**, the anion was involved in Se···X and H···X bifurcated non-covalent interactions (Figure 1). This robust chalcogen-bonded supramolecular synthon was described by us earlier [44,45,46]. Importantly, all the *α*-haloalkyl-substituted pyridinium-fused 1,2,4-selenadiazoles **3**–**10** exclusively form supramolecular dimers via four-center Se···N ChB (Figure 1) without an exception. Trichloromethyl-substituted 1,2,4-selenadiazoles **3** and **4** feature two antiparallel XB interactions Cl···Cl (for **3**) or Cl···OReO_3_ (for **4**, Figure 1). Chloromethyl-substituted derivatives **5** (PF_6_ salt) and **6** (BF_4_ salt) apart from four-center Se···N ChB exhibit Cl···F XB and Se···F ChB. 2,2-Dibromo-2-cyanoacetamide-derived pyridinium-fused 1,2,4-selenadiazole bromide **7** also exhibited four-center Se···N ChB together with the peripheral Br···Br interactions (Figure 1).

Further, we prepared fluoromethyl-substituted 1,2,4-selenadiazole salts **8** and **9** (Figure 1). Interestingly, they also formed dimers via four-center Se···N ChB but did not form F···X XB with the anions. In these cases, H···X HB (Figure 1) outcompeted the formation of XB involving the fluorine atom, arguably due to the low polarizability of the F atom and its weak XB-donating ability.

Finally, thiocyanate salt **10** also self-assembled into antiparallel supramolecular dimers in the solid state via four-center Se···N ChB and a pair of Cl···NCS XB (Figure 1). 

Thus, the introduction of a halogen at the α-position of substituent R in the selenadiazole core indeed promotes supramolecular dimerization via four-center Se···N ChB; anion variation does not break these robust dimers as demonstrated above. 

**Scheme 2:**
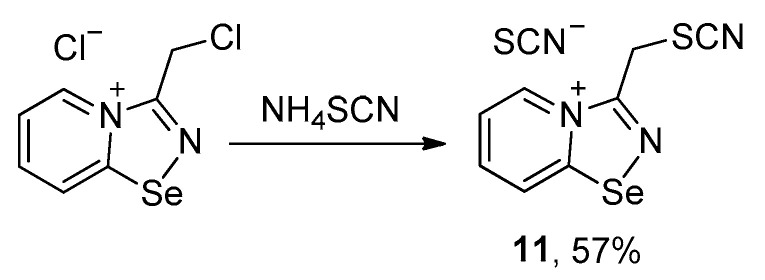
Synthesis of **11**.

Further, we aimed to obtain thiocyanate salt of chloromethyl-substituted 1,2,4-selenadiazole salt via anion metathesis, but obtained thiocyano-substituted derivative **11** due to chlorine-to-thiocyanate exchange (Scheme 2).

**Figure 2:**
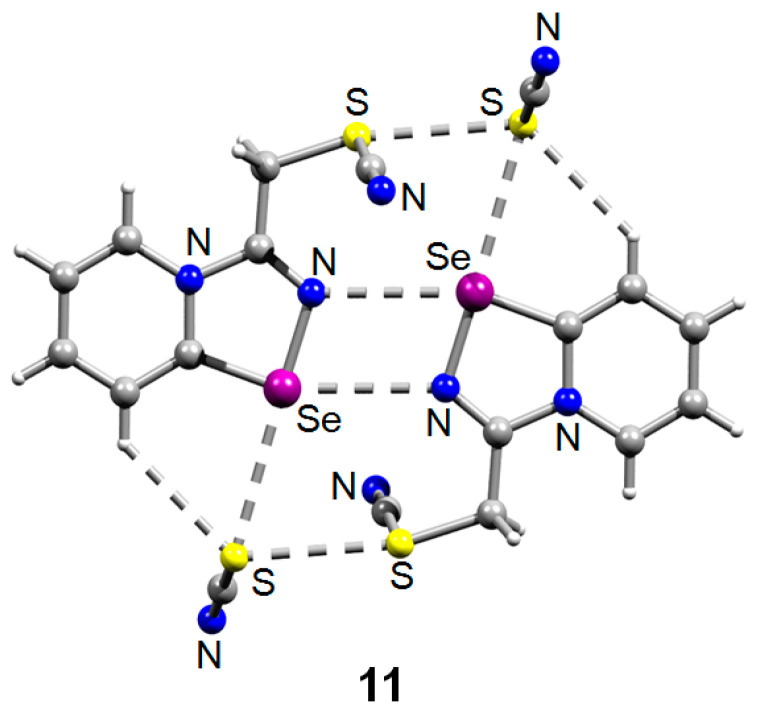
Ball-and-stick representation of the crystal structure of **11** demonstrating supramolecular dimerization via four center Se···N ChB assisted by the S···S ChB. Grey and light-grey spheres represent carbon and hydrogen, respectively.

The reaction was reproducible and allowed the preparation of **11** in good yield (57%). We managed to obtain single crystals suitable for the X-ray structural analysis, which revealed that **11** also self-assembles in the solid into Se_2_N_2_ supramolecular dimers, which are supported by a pair of S···S ChB interactions (Figure 2).

Further, we were interested in how cocrystallization of α-haloalkyl-substituted pyridinium-fused 1,2,4-selenadiazoles with C_6_F_3_I_3_, which is a classical halogen bond donor, would affect the self-assembly of a resulting supramolecular aggregate and whether Se_2_N_2_ supramolecular dimers would be sustained.

**Figure 3:**
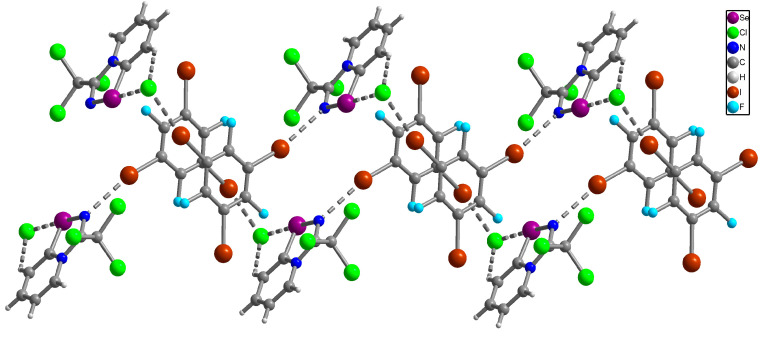
Ball-and-stick representation of the crystal structure of **12** demonstrating Se···Cl ChB, I···N, I···Cl XB and H···Cl HB. Purple, blue, green, brown, cyan, grey and light-grey spheres represent selenium, nitrogen, chlorine, iodine, fluorine, carbon and hydrogen, respectively.

For this reason, we cocrystallized α-(trichloromethyl)-[1,2,4]selenadiazolo [4,5-a]pyridin-4-ium chloride with C_6_F_3_I_3_ (1:1 ratio) using MeOH as a solvent and performed single crystal structural analysis for a cocrystal **12** (Figure 3).

X-ray analysis revealed that in the solid of **12** Se_2_N_2_ supramolecular dimers are broken (Se···N distances of 5.82 Å are too long for ChB). However, Se···Cl and H···Cl bifurcated non-covalent interactions between the heterocycle and the Cl anion are conserved demonstrating again the robustness of this supramolecular synthon. Moreover, **12** contains supramolecular 1D infinite chains consisting of alternating selenadiazole···Cl ion pairs and C_6_F_3_I_3_ molecules (Figure 3), which are interconnected by I···N and I···Cl XB. Thus, in the resulting solid **12** Se_2_N_2_ supramolecular dimers were disrupted, indicating that the formed I···N and I···Cl XB interactions involving C_6_F_3_I_3_ molecules were collectively more significant than ChBs and HBs, which is confirmed by the results of DFT calculations and topological analysis of the electron density distribution within the framework of Bader’s theory (QTAIM analysis) [47] for model supramolecular associates (see properties and estimated strengths of such contacts in Table 1 and Table 2).

**Figure 4:**
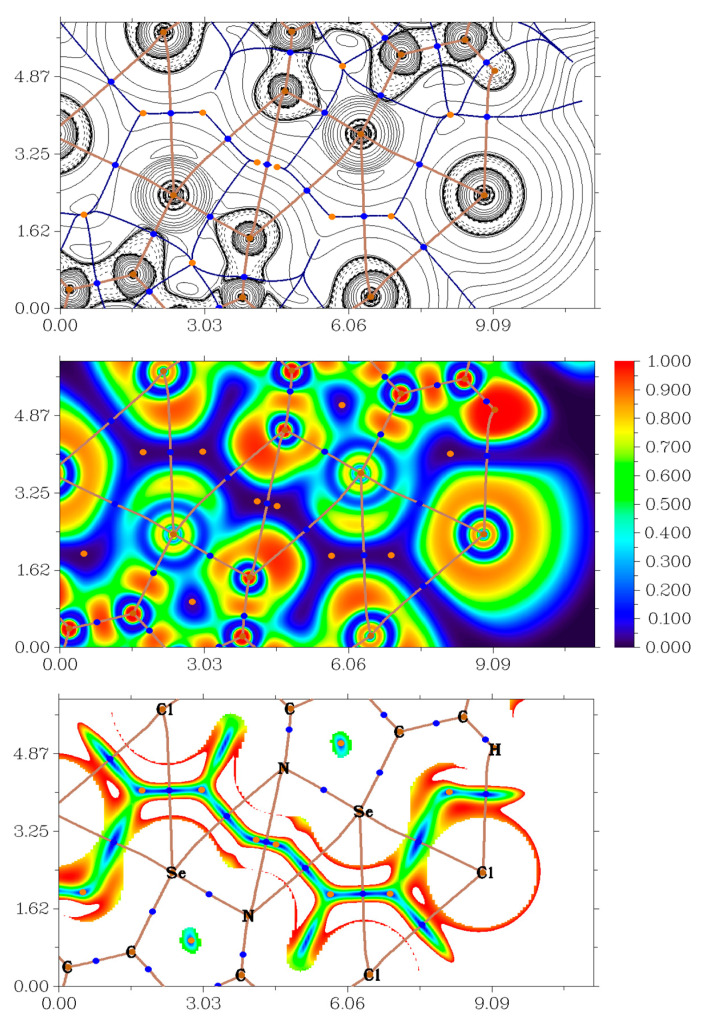
Contour line diagram of the Laplacian of electron density distribution ∇^2^ρ(**r**), bond paths and selected zero-flux surfaces (**top panel**), visualization of electron localization function (ELF, **center panel**) and reduced density gradient (RDG, **bottom panel**) analyses for H···Cl, Se···N, Se···C and Cl···Cl non-covalent interactions in **3**. Bond critical points (3, −1) are shown in blue, nuclear critical points (3, −3) in pale brown, ring critical points (3, +1) in orange, bond paths are shown as pale brown lines, length units on axis is Å and the color scale for the ELF and RDG maps is presented in a.u.

In order to confirm the presence of discussed HB, XB and ChB in studied solids **3**–**12** from a theoretical viewpoint, we carried out DFT calculations at the ωB97XD/DZP-DKH level of theory followed by the topological analysis of the electron density distribution within the framework of Bader’s theory (QTAIM analysis) [47] for model supramolecular associates (Cartesian atomic coordinates for these model supramolecular associates are presented in Supplementary Materials). The results of QTAIM analysis are summarized in Table 1 and Table 2; for illustrative purposes, the contour line diagram of the Laplacian of electron density distribution ∇^2^ρ(**r**), bond paths and selected zero-flux surfaces, visualization of electron localization function (ELF) and reduced density gradient (RDG) analyses for H···Cl, Se···N, Se···Cl, and Cl···Cl non-covalent interactions in **3** are shown in Figure 4.

The QTAIM analysis demonstrates the presence of appropriate bond critical points (3, −1) for HB, XB and ChB in model supramolecular associates (Table 1). The low magnitude of the electron density, positive values of the Laplacian of electron density and zero or very close to zero positive energy density in these bond critical points (3, −1) and estimated strengths for appropriate short contacts are typical for such non-covalent interactions is similar chemical systems [38,41,48,49,50,51]. The balance between the Lagrangian kinetic energy G(**r**) and potential energy density V(**r**) at the bond critical points (3, −1) corresponding for HB, XB and ChB in model supramolecular associates reveals that a covalent contribution is absent in these contacts (−G(**r**)/V(**r**) > 1) [52]. The sign of λ_2_ can be utilized to distinguish bonding (attractive, λ_2_ < 0) weak interactions from nonbonding ones (repulsive, λ_2_ > 0) [4,53,54]. Thus, discussed non-covalent interactions are attractive (Table 1).

## 3. Materials and Methods

### 3.1. General Remarks

All manipulations were carried out in the air. All the reagents used in this study were obtained from commercial sources (Aldrich, TCI-Europe, Strem, ABCR). Commercially available solvents were purified by conventional methods and distilled immediately prior to use. NMR spectra were recorded on a Bruker Avance NEO 700 (Karlsruhe, Germany); chemical shifts (*δ*) are given in ppm and coupling constants (*J*) in Hz. 4,4′-Dimethyl-2,2′-dipyridyl diselenide was obtained by the method reported in [55].

### 3.2. X-ray Crystal Structure Determination

The single-crystal X-ray diffraction data were collected on a three-circle Bruker D8 Venture diffractometer (Karlsruhe, Germany) (graphite monochromator, *w* and *φ* scan mode) (3, 4, 6–9, 11), on the ‘Belok/RSA’ beamline of the National Research Center ‘Kurchatov Institute’ (Moscow, Russian Federation) using a Rayonix SX165 CCD detector (Evanston, IL USA) (*φ* scan mode) (5) and on a four-circle Rigaku Synergy S diffractometer equipped with a HyPix6000HE area-detector (Tokyo, Japan) (graphite monochromator, shutterless *ω* scan mode) (10, 12). For compounds **3**, **4**, **6**–**9** and **11**, the data were indexed and integrated using the *SAINT* program [56] and then scaled and corrected for absorption using the *SADABS* program [57]. For compound **5**, the data were integrated by the utility *iMOSFLM* in the CCP4 program [58] and corrected for absorption using the *Scala* program [59]. For compounds **10** and **12**, the data were integrated and corrected for absorption by the *CrysAlisPro* program (Rigaku, *CrysAlisPro Software System, v. 1.171.41.106a*, Rigaku Oxford Diffraction, 2021). For details, see Table S1 (electronic Supporting Information). The structures were determined by direct methods and refined by full-matrix least squares technique on *F*^2^ with anisotropic displacement parameters for non-hydrogen atoms. The amino hydrogen atoms in 7 were localized in the difference-Fourier maps and refined within the riding model with fixed isotropic displacement parameters [*U*_iso_(H) = 1.2*U*_eq_(N)]. The other hydrogen atoms in all compounds were placed in calculated positions and refined within the riding model with fixed isotropic displacement parameters [*U*_iso_(H) = 1.5*U*_eq_(C) for the CH_3_ groups and 1.2*U*_eq_(C) for the other groups]. All calculations were carried out using the SHELXTL program suite [60].

Crystallographic data for compounds **3**–**12** have been deposited into the Cambridge Crystallographic Data Center, CCDC 2341614-2341623, respectively. Copies of this information may be obtained free of charge from the Director, CCDC, 12 Union Road, Cambridge CHB2 1EZ, UK (fax: +44 1223 336033; e-mail: deposit@ccdc.cam.ac.uk or www.ccdc.cam.ac.uk).

### 3.3. Computational Details

The single-point calculations based on the experimental X-ray structures **3**–**12** have been carried out at the DFT level of theory using the dispersion-corrected hybrid functional ωB97XD [61] with the help of the Gaussian 09 [62] program package. The Douglas–Kroll–Hess 2nd order scalar relativistic calculations requested relativistic core Hamiltonian were carried out using the DZP-DKH basis sets [63] for all atoms. The topological analysis of the electron density distribution with the help of the atoms in molecules (QTAIM) method has been performed by using the Multiwfn program (version 3.7) [64]. The Cartesian atomic coordinates for model supramolecular associates are presented in the Supplementary Materials.

### 3.4. Synthesis of Compounds ***3***–***11***


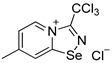


A solution of PhICl_2_ (88 mg, 320 μmol) in CH_2_Cl_2_ (2 mL) was added to a solution of 4,4′-dimethyl-2,2′-dipyridyldiselenide (100 mg, 292 μmol) in Et_2_O (5 mL), and the reaction mixture was allowed to stand without stirring at room temperature for 12 h. Subsequently, the solution was separated from a yellow precipitate, and the solid was washed with Et_2_O (3 × 1 mL) and dried under a vacuum. Yield: 52 mg (43%). ^1^H NMR (600 MHz, CDCl_3_) *δ* 8.48 (d, *J* = 6.0 Hz, 1H), 8.37 (d, *J* = 6.0 Hz, 1H), 7.97 (s, 1H), 2.49 (s, 3H). ^13^C NMR (151 MHz, CDCl_3_) *δ* 139.6, 22.0.


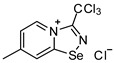


**3**. A solution of PhICl_2_ (27 mg, 98 μmol) in CH_2_Cl_2_ (2 mL) was added to a solution of 4,4′-dimethyl-2,2′-dipyridyldiselenide (30 mg, 88 μmol) and trichloroacetonitrile (50 μL, 499 μmol) in CH_2_Cl_2_ (2 mL), and the reaction mixture was left without stirring at room temperature for 12 h. After that, the solution was decanted from a colorless precipitate, and the solid was washed with Et_2_O (3 × 1 mL) and dried under a vacuum. Yield: 48 mg (78%). ^1^H NMR (600 MHz, D_2_O) *δ* 9.75 (d, *J* = 7.2 Hz, 1H), 8.69 (s, 1H), 7.95 (dd, *J* = 7.2, 1.8 Hz, 1H), 2.73 (s, 3H). ^13^C NMR (151 MHz, D_2_O) *δ* 170.2, 155.6, 147.8, 137.0, 125.7, 124.9, 87.5, 21.6.


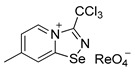


**4**. 7-methyl-3-(trichloromethyl)-[1,2,4]selenadiazolo [4,5-a]pyridin-4-ium chloride 3 (15 mg, 43 μmol) was dissolved in MeOH (1.5 mL) and the addition of 20 μL of perrhenic acid (70 wt %) resulted in the formation of a colorless microcrystalline precipitate, which was washed with Et_2_O (3 × 3 mL) and dried in vacuum. Yield: 10 mg (42%). ^1^H NMR (700 MHz, DMSO-*d*_6_) *δ* 9.68 (d, *J* = 7.1 Hz, 1H), 8.83–8.82 (m, 1H), 7.99–7.95 (m, 1H), 2.71 (s, 3H). ^13^C NMR (176 MHz, DMSO-*d*_6_) *δ* 172.2, 153.9, 146.8, 137.1, 126.7, 125.2, 88.7, 22.2.


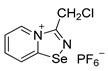


**5**. 3-(chloromethyl)-[1,2,4]selenadiazolo[4,5-a]pyridin-4-ium chloride (15 mg, 56 μmol) was dissolved in MeOH (1.5 mL) and the addition of the saturated MeOH solution of NBu_4_PF_6_ (300 µL) resulted in the formation of colorless crystals, which were washed with Et_2_O (3×3 mL) and dried under vacuum. Yield: 9 mg (43%). ^1^H NMR (600 MHz, D_2_O) *δ* 9.51 (d, *J* = 6.8 Hz, 1H), 8.86 (d, *J* = 8.7 Hz, 1H), 8.48–8.43 (m, 1H), 8.10–8.07 (m, 1H), 5.34 (s, 2H). ^13^C NMR (151 MHz, D_2_O) *δ* 168.8, 153.0, 140.0, 136.5, 126.2, 123.4, 37.8.


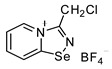


**6**. 3-(chloromethyl)-[1,2,4]selenadiazolo[4,5-a]pyridin-4-ium chloride (15 mg, 56 μmol) was dissolved in MeOH (1.5 mL) and the addition of 10 μL of HBF_4_ (40 wt %) resulted in the formation of yellow crystals, which were washed with Et_2_O (3 × 3 mL) and dried under a vacuum. Yield: 8 mg (48%). ^1^H NMR (600 MHz, D_2_O) *δ* 9.50 (d, *J* = 6.8 Hz, 1H), 8.85 (d, *J* = 8.7 Hz, 1H), 8.45 (t, *J* = 8.3 Hz, 1H), 8.08 (t, *J* = 7.4 Hz, 1H), 5.34 (s, 2H). ^13^C NMR (151 MHz, D_2_O) *δ* 168.8, 153.0, 139.9, 136.4, 126.2, 123.4, 37.8.


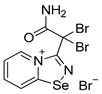


**7**. A solution of bromine (15 mg, 96 μmol) in CH_2_Cl_2_ (1 mL) was added to a solution of 2,2′-dipyridyldiselenide (30 mg, 96 μmol) and 2,2-dibromo-2-cyanoacetamide (46 mg, 192 μmol) in CH_2_Cl_2_ (2 mL), and the reaction mixture was left without stirring at room temperature for 12 h. After that, the solution was decanted from a yellow precipitate, and the solid was washed with Et_2_O (3×1 mL) and dried under a vacuum. Yield: 73 mg (79%). ^1^H NMR (600 MHz, D_2_O) *δ* 9.41 (d, *J* = 6.8 Hz, 1H), 8.92 (d, *J* = 8.7 Hz, 1H), 8.48–8.43 (m, 1H), 8.08 (t, *J* = 7.1 Hz, 1H). ^13^C NMR (151 MHz, D_2_O) *δ* 171.2, 167.0, 149.1, 140.0, 137.7, 126.7, 123.2, 47.1.


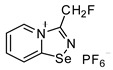


**8**. 3-(fluoromethyl)-[1,2,4]selenadiazolo[4,5-a]pyridin-4-ium chloride (15 mg, 59.6 μmol) was dissolved in MeOH (1.5 mL) and addition of the saturated MeOH solution of NBu_4_PF_6_ (300 µL) resulted in the formation of colorless crystals, which were washed with Et_2_O (3 × 3 mL) and dried under a vacuum. Yield: 9 mg (42%). ^1^H NMR (700 MHz, D_2_O) *δ* 9.47 (d, *J* = 6.8 Hz, 1H), 8.86 (d, *J* = 8.7 Hz, 1H), 8.46 (t, *J* = 8.0 Hz, 1H), 8.08 (t, *J* = 7.0 Hz, 1H), 6.11 (s, 1H), 6.04 (s, 1H). ^13^C NMR (176 MHz, D_2_O) *δ* 168.6, 152.4, 140.0, 136.3, 126.1, 123.4, 78.5, 77.5.


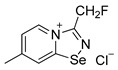


**9**. A solution of PhICl_2_ (27 mg, 98 μmol) in CH_2_Cl_2_ (2 mL) was added to a solution of 4,4′-dimethyl-2,2′-dipyridyldiselenide (30 mg, 88 μmol) and fluoroacetonitrile (50 μL, 890 μmol) in CH_2_Cl_2_ (2 mL), and the reaction mixture was left without stirring at room temperature for 12 h. After that, the solution was decanted from a colorless precipitate, and the solid was washed with Et_2_O (3 × 1 mL) and dried under a vacuum. Yield: 34 mg (72%). ^1^H NMR (600 MHz, D_2_O) *δ* 9.26 (d, *J* = 6.9 Hz, 1H), 8.61–8.60 (m, 1H), 7.89 (dd, *J* = 7.0, 1.6 Hz, 1H), 6.05 (s, 1H), 5.97 (s, 1H), 2.70 (s, 3H). ^13^C NMR (151 MHz, D_2_O) *δ* 167.3, 155.3, 135.0, 125.4, 125.3, 78.6, 77.5, 21.6.


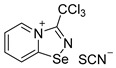


**10**. 3-(trichloromethyl)-[1,2,4]selenadiazolo[4,5-a]pyridin-4-ium chloride (15 mg, 44.5 μmol) was dissolved in MeOH (1.5 mL) and the addition of the saturated MeOH solution of NH_4_SCN (100 µL) resulted in the formation of colorless crystals, which were washed with EtOH (3 × 3 mL) and dried under a vacuum. Yield: 7 mg (44%). ^1^H NMR (700 MHz, D_2_O) *δ* 9.37 (dt, *J* = 6.8, 1.0 Hz, 1H), 8.80 (dt, *J* = 8.7, 1.0 Hz, 1H), 8.40–8.36 (m, 1H), 8.02–7.99 (m, 1H). ^13^C NMR (176 MHz, D_2_O) *δ* 171.6, 148.1, 140.1, 138.3, 133.1, 126.4, 123.1, 87.5.


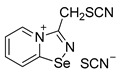


**11**. 3-(chloromethyl)-[1,2,4]selenadiazolo[4,5-a]pyridin-4-ium chloride (15 mg, 56 μmol) was dissolved in MeOH (1.5 mL) and addition of the saturated MeOH solution of NH_4_SCN (100 µL) resulted in the formation of colorless crystals, which were washed with EtOH (3 × 3 mL) and dried under a vacuum. Yield: 10 mg (57%). ^1^H NMR (700 MHz, D_2_O) *δ* 9.50 (dd, *J* = 12.6, 6.8 Hz, 1H), 8.86 (d, *J* = 8.7 Hz, 1H), 8.46 (t, *J* = 7.9 Hz, 1H), 8.09 (q, *J* = 6.8 Hz, 1H), 5.35 (s, 1H), 5.18 (s, 1H). ^13^C NMR (176 MHz, D_2_O) *δ* 168.8, 151.9, 139.9, 135.8, 133.5, 126.3, 123.4, 112.2, 31.9.

## 4. Conclusions

Overall, we prepared and structurally characterized eight α-haloalkyl-substituted pyridinium-fused 1,2,4-selenadiazoles with various counterions. Our findings demonstrate that incorporating a halogen at the α-position of the R substituent in the selenadiazole core proves to be an effective strategy for inducing directed supramolecular dimerization of selenadiazoles in the solid state.

Across all cases, the Se_2_N_2_ supramolecular motif was consistently supported by two symmetrically equivalent halogen–anion (XB) interactions, with hydrogen bonding (HB) also playing a crucial role in the self-assembly and supramolecular organization of these chemical systems in the solid state. Furthermore, we investigated how the cocrystallization of α-haloalkyl-substituted pyridinium-fused 1,2,4-selenadiazoles with C_6_F_3_I_3_ would affect the self-assembly of a resulting supramolecular aggregate.

In the resulting solid Se_2_N_2_ supramolecular dimers were disrupted, indicating that the formed I···N and I···Cl XB interactions involving C_6_F_3_I_3_ were collectively more significant than ChB. Considering the fundamental role of ChB, XB and HB interactions in the crystal packing of studied solids **3**–**12**, these intermolecular contacts were also investigated theoretically.

Results of DFT calculations and topological analysis of the electron density distribution in model supramolecular associates reveal that all ChB, XB and HB interactions are purely non-covalent and attractive in nature. Overall, the estimated strength of these weak contacts decreases in the following order: 1.6–6.3 kcal/mol (ChB), 0.9–4.1 kcal/mol (XB) and 0.6–2.8 kcal/mol (HB).

Hence, halogen bond-assisted supramolecular dimerization of pyridinium-fused 1,2,4-selenadiazoles via four-center Se_2_N_2_ chalcogen bonding emerges as a potent tool in crystal engineering. We anticipate that this approach will find widespread adoption by researchers in the future for creating extended molecular systems connected via non-covalent interactions.

## Figures and Tables

**Table 1:** Values of the density of all electrons—ρ(**r**), Laplacian of electron density—∇^2^ρ(**r**) and appropriate λ_2_ eigenvalues, energy density—H_b_, potential energy density—V(**r**), and Lagrangian kinetic energy—G(**r**) (a.u.) at the bond critical points (3, −1) corresponding with hydrogen, halogen and chalcogen bonding in studied crystal structures **3**–**12**.

Contact	ρ(r)	∇^2^ρ(r)	λ_2_	H_b_	V(r)	G(r)
**3**						
Se···N 3.199 Å	0.010	0.033	−0.010	0.001	−0.006	0.007
Se···Cl 2.886 Å	0.027	0.071	−0.027	0.000	−0.017	0.017
Cl···Cl 3.201 Å	0.012	0.041	−0.012	0.002	−0.007	0.009
H···Cl 2.590 Å	0.009	0.039	−0.009	0.003	−0.005	0.008
**4**						
Se···N 3.139 Å	0.011	0.037	−0.011	0.002	−0.006	0.008
Se···O 2.611 Å	0.026	0.101	−0.026	0.003	−0.020	0.023
Cl···O 3.334 Å	0.006	0.022	−0.006	0.001	−0.003	0.004
Cl···O 3.180 Å	0.007	0.029	−0.007	0.001	−0.005	0.006
Cl···Se 3.644 Å	0.006	0.018	−0.006	0.001	−0.003	0.004
H···O 2.473 Å	0.010	0.043	−0.010	0.002	−0.007	0.009
**5**						
Se···N 2.951 Å	0.015	0.055	−0.015	0.002	−0.010	0.012
Se···F 2.916 Å	0.012	0.049	−0.012	0.002	−0.008	0.010
Cl···F 3.153 Å	0.006	0.027	−0.006	0.001	−0.004	0.005
Cl···F 3.286 Å	0.005	0.020	−0.005	0.001	−0.003	0.004
H···F 2.441 Å	0.008	0.033	−0.008	0.001	−0.006	0.007
H···F 2.633 Å	0.006	0.026	−0.006	0.001	−0.004	0.005
**6**						
Se···N 2.981 Å	0.014	0.052	−0.014	0.002	−0.009	0.011
Se···F 2.902 Å	0.012	0.050	−0.012	0.002	−0.008	0.010
Cl···F 3.312 Å	0.004	0.018	−0.004	0.001	−0.003	0.004
Cl···F 3.293 Å	0.005	0.020	−0.005	0.001	−0.003	0.004
H···F 2.345 Å	0.010	0.038	−0.010	0.001	−0.007	0.008
H···F 2.575 Å	0.006	0.029	−0.006	0.001	−0.005	0.006
**7**						
Se···N 3.125 Å	0.011	0.039	−0.011	0.002	−0.007	0.008
Se···Br 3.230 Å	0.010	0.028	−0.010	0.001	−0.005	0.006
Br···Br 3.220 Å	0.017	0.042	−0.017	0.000	−0.010	0.010
Br···Br 3.316 Å	0.016	0.032	−0.016	0.000	−0.008	0.008
H···Br 2.716 Å	0.011	0.041	−0.011	0.001	−0.008	0.009
**8**						
Se···N 2.892 Å	0.017	0.062	−0.017	0.002	−0.011	0.013
Se···F 2.953 Å	0.012	0.045	−0.012	0.002	−0.007	0.009
Se···F 3.068 Å	0.010	0.037	−0.010	0.002	−0.006	0.008
H···F 2.583 Å	0.006	0.029	−0.006	0.002	−0.004	0.006
H···F 2.768 Å	0.004	0.015	−0.004	0.001	−0.002	0.003
H···F 2.838 Å	0.003	0.013	−0.003	0.001	−0.002	0.003
**9**						
Se···N 3.029 Å	0.013	0.047	−0.013	0.002	−0.008	0.010
Se···Cl 2.968 Å	0.024	0.062	−0.024	0.001	−0.014	0.015
H···Cl 2.712 Å	0.011	0.037	−0.011	0.001	−0.007	0.008
**10**						
Se···N 3.239 Å	0.009	0.030	−0.009	0.001	−0.005	0.006
Se···N 2.694 Å	0.025	0.079	−0.025	0.001	−0.017	0.018
Cl···C 3.197 Å	0.007	0.029	−0.007	0.002	−0.004	0.006
H···N 2.422 Å	0.012	0.048	−0.012	0.002	−0.008	0.010
**11**						
Se···N 3.101 Å	0.012	0.040	−0.012	0.002	−0.007	0.009
Se···S 3.591 Å	0.008	0.025	−0.008	0.001	−0.004	0.005
Se···C 3.402 Å	0.007	0.024	−0.007	0.001	−0.004	0.005
Se···S 3.201 Å	0.018	0.042	−0.018	0.001	−0.008	0.009
H···S 2.922 Å	0.008	0.025	−0.008	0.001	−0.004	0.005
**12**						
I···Cl 3.358 Å	0.013	0.043	−0.013	0.001	−0.009	0.010
I···Cl 3.347 Å	0.014	0.044	−0.014	0.001	−0.009	0.010
I···Cl 3.353 Å	0.014	0.044	−0.014	0.001	−0.009	0.010
I···Cl 3.180 Å	0.019	0.055	−0.019	0.000	−0.013	0.013
I···N 3.116 Å	0.015	0.052	−0.015	0.001	−0.011	0.012
Se···Cl 2.968 Å	0.023	0.064	−0.023	0.001	−0.014	0.015
H···Cl 2.583 Å	0.014	0.046	−0.014	0.001	−0.009	0.010

**Table 2:** Estimated binding energies (E_int_, kcal/mol) of HB, XB and ChB in studied crystal structures **3**–**12**.

Contact	E_int_ ≈ –V(r)/2
**3**	
Se···N 3.199 Å	1.9
Se···Cl 2.886 Å	5.3
Cl···Cl 3.201 Å	2.2
H···Cl 2.590 Å	1.6
**4**	
Se···N 3.139 Å	1.9
Se···O 2.611 Å	6.3
Cl···O 3.334 Å	0.9
Cl···O 3.180 Å	1.6
Cl···Se 3.644 Å	0.9
H···O 2.473 Å	2.2
**5**	
Se···N 2.951 Å	3.1
Se···F 2.916 Å	2.5
Cl···F 3.153 Å	1.3
Cl···F 3.286 Å	0.9
H···F 2.441 Å	1.9
H···F 2.633 Å	1.3
**6**	
Se···N 2.981 Å	2.8
Se···F 2.902 Å	2.5
Cl···F 3.312 Å	0.9
Cl···F 3.293 Å	0.9
H···F 2.345 Å	2.2
H···F 2.575 Å	1.6
**7**	
Se···N 3.125 Å	2.2
Se···Br 3.230 Å	1.6
Br···Br 3.220 Å	3.1
Br···Br 3.316 Å	2.5
H···Br 2.716 Å	2.5
**8**	
Se···N 2.892 Å	3.5
Se···F 2.953 Å	2.2
Se···F 3.068 Å	1.9
H···F 2.583 Å	1.3
H···F 2.768 Å	0.6
H···F 2.838 Å	0.6
**9**	
Se···N 3.029 Å	2.5
Se···Cl 2.968 Å	4.4
H···Cl 2.712 Å	2.2
**10**	
Se···N 3.239 Å	1.6
Se···N 2.694 Å	5.3
Cl···C 3.197 Å	1.3
H···N 2.422 Å	2.5
**11**	
Se···N 3.101 Å	2.2
Se···S 3.591 Å	1.3
Se···C 3.402 Å	1.3
Se···S 3.201 Å	2.5
H···S 2.922 Å	1.3
**12**	
I···Cl 3.358 Å	2.8
I···Cl 3.347 Å	2.8
I···Cl 3.353 Å	2.8
I···Cl 3.180 Å	4.1
I···N 3.116 Å	3.5
Se···Cl 2.968 Å	4.4
H···Cl 2.583 Å	2.8

## Data Availability

Data are contained within the article and Supplementary Materials.

## Supplementary Materials

The following supporting information can be downloaded at: https://www.mdpi.com/article/10.3390/ijms25073972/s1.
