# Supplementary material for: Halogen Bond-Assisted Supramolecular Dimerization of Pyridinium-Fused 1,2,4-Selenadiazoles via Four-Center Se2N2 Chalcogen Bonding
Source: Int J Mol Sci. 2024 Apr 3;25(7):3972. doi: 10.3390/ijms25073972 (PMC11011651; doi:10.3390/ijms25073972)
Supplement: Supplementary file 1 [file ijms-25-03972-s001.zip › ijms-2949782-supplementary.pdf]

Table S1. Crystal data and structure refinement for **3-12**.

Identification code	**3** • CH_2Cl_2	**4**	**5**	**6**
Empirical formula	$C_9H_8Cl_6N_2Se$	$C_8H_6Cl_3N_2O_4ReSe$	$C_7H_6ClF_6N_2PSe$	$C_7H_6BClF_4N_2Se$
Formula weight	435.83	565.67	377.52	319.36
Temperature/K	100	150	100	100
Crystal system	monoclinic	monoclinic	monoclinic	monoclinic
Space group	$P2_1/n$	$P2_1/c$	$C2/c$	$C2/c$
a/Å	6.7342(12)	7.376(3)	14.5234(12)	7.3497(15)
b/Å	29.004(5)	16.205(6)	7.7407(6)	14.196(3)
c/Å	8.1255(16)	12.277(4)	21.4610(18)	20.748(4)
α/°	90	90	90	90
β/°	113.843(10)	106.768(13)	98.008(8)	91.812(12)
γ/°	90	90	90	90
Volume/Å^3	1451.6(5)	1405.1(8)	2389.1(3)	2163.7(8)
Z	4	4	8	8
d_{calc}, g/cm^3	1.994	2.674	2.099	1.961
μ/mm^{-1}	3.672	11.817	4.020	3.743
F(000)	848	1040	1456	1232
Radiation/Å	Mo$K\alpha$ (λ = 0.71073)	Mo$K\alpha$ (λ = 0.71073)	Synchrotron (λ = 0.74500)	Mo$K\alpha$ (λ = 0.71073)
Θ range for data collection/°	2.106 to 25.248	2.140 to 30.019	3.226 to 31.023	1.964 to 25.249
Reflections collected	4987	8797	18406	7191
Independent reflections	2420 [R_{int} = 0.0538]	4005 [R_{int} = 0.0433]	3285 [R_{int} = 0.0274]	1906 [R_{int} = 0.0525]
Reflections observed [I>=2σ(I)]	2152	3131	3051	1806
Final R indexes [I>=2σ (I)]	R_1 = 0.1099, wR_2 = 0.2596	R_1 = 0.0459, wR_2 = 0.0764	R_1 = 0.0361, wR_2 = 0.1002	R_1 = 0.1090, wR_2 = 0.2408
Final R indexes [all data]	R_1 = 0.1202, wR_2 = 0.2662	R_1 = 0.0666, wR_2 = 0.0846	R_1 = 0.0380, wR_2 = 0.1017	R_1 = 0.1134, wR_2 = 0.2450
Goodness-of-fit on F^2	1.096	1.115	1.093	1.039
T_{min} / T_{max}	0.337 / 0.746	0.192 / 0.444	0.567 / 0.654	0.477 / 0.725
$\Delta\rho_{max}$ / $\Delta\rho_{min}$, e·Å^{-3}	2.224 / -1.649	1.854 / -1.826	1.383 / -0.654	3.541 / -1.394

Identification code	**7**	**8**	**9**	**10**
Empirical formula	$C_8H_6Br_3N_3OSe$	$C_7H_6F_7N_2PSe$	$C_8H_8ClFN_2Se$	$C_8H_4Cl_3N_3SSe$
Formula weight	478.82	361.07	265.57	359.51
Temperature/K	100	100	100	100
Crystal system	monoclinic	tetragonal	triclinic	monoclinic
Space group	$P2_1$/n	$I4_1$cd	*P*-1	$P2_1$/n
a/Å	11.7081(11)	17.1433(3)	6.9873(11)	7.66749(4)
b/Å	9.94400(10)	17.1433(3)	7.3717(13)	11.53184(7)
c/Å	12.0539(12)	15.4180(4)	9.3377(16)	13.73130(8)
α/°	90	90	99.810(10)	90
β/°	117.250(9)	90	92.829(9)	92.0444(5)
γ/°	90	90	97.834(9)	90
Volume/Å^3	1247.6(2)	4531.2(2)	468.21(14)	1213.353(12)
Z	4	16	2	4
d_{calc}, g/cm^3	2.549	2.117	1.884	1.968
μ/mm^{-1}	12.599	3.532	4.261	11.680
F(000)	888	2784	260	696
Radiation/Å	Mo$K\alpha$ (λ = 0.71073)	Mo$K\alpha$ (λ = 0.71073)	Mo$K\alpha$ (λ = 0.71073)	Cu$K\alpha$ (λ = 1.54184)
Θ range for data collection/°	2.009 to 28.449	2.376 to 34.038	2.220 to 30.060	5.010 to 79.910
Reflections collected	7271	26057	3562	19715
Independent reflections	3134 [R_{int} = 0.0472]	4260 [R_{int} = 0.0466]	2611 [R_{int} = 0.0204]	2628 [R_{int} = 0.0312]
Reflections observed [I>=2σ(I)]	2238	3449	2267	2608
Final R indexes [I>=2σ (I)]	R_1 = 0.0389, wR_2 = 0.0721	R_1 = 0.0307, wR_2 = 0.0559	R_1 = 0.0348, wR_2 = 0.0840	R_1 = 0.0208, wR_2 = 0.0509
Final R indexes [all data]	R_1 = 0.0719, wR_2 = 0.0826	R_1 = 0.0512, wR_2 = 0.0605	R_1 = 0.0434, wR_2 = 0.0886	R_1 = 0.0210, wR_2 = 0.0510
Goodness-of-fit on F^2	1.044	1.025	1.041	1.140
T_{min} / T_{max}	0.046 / 0.276	0.470 / 0.545	0.638 / 0.826	0.201 / 0.543
$\Delta\rho_{max}$ / $\Delta\rho_{min}$, e·Å^{-3}	1.168 / -1.063	0.455 / -0.778	0.749 / -0.897	0.438 / -0.334

Identification code	**11**	**12** • $2C_6F_3I_3$
Empirical formula	$C_9H_6N_4S_2Se$	$C_{19}H_4Cl_4F_6I_6N_2Se$
Formula weight	313.26	1356.40
Temperature/K	100	100
Crystal system	monoclinic	triclinic
Space group	$P2_1/n$	P-1
a/Å	7.310(2)	9.47224(11)
b/Å	10.024(4)	11.7283(2)
c/Å	15.069(5)	16.0719(3)
α/°	90	72.8369(16)
β/°	92.168(12)	75.6502(12)
γ/°	90	66.2987(15)
Volume/Å^3	1103.4(6)	2164.6(3)
Z	4	2
d_{calc}, g/cm^3	1.886	2.916
μ/mm^{-1}	3.756	52.391
F(000)	616	1212
Radiation/Å	Mo$K\alpha$ (λ = 0.71073)	Cu$K\alpha$ (λ = 1.54184)
Θ range for data collection/°	2.441 to 29.306	2.910 to 79.935
Reflections collected	9097	40328
Independent reflections	2998 [R_{int} = 0.0454]	6626 [R_{int} = 0.0972]
Reflections observed [I>=2σ(I)]	2292	6503
Final R indexes [I>=2σ (I)]	R_1 = 0.0372, wR_2 = 0.0738	R_1 = 0.0596, wR_2 = 0.1368
Final R indexes [all data]	R_1 = 0.0593, wR_2 = 0.0852	R_1 = 0.0603, wR_2 = 0.1371
Goodness-of-fit on F^2	1.035	1.004
T_{min} / T_{max}	0.509 / 0.746	0.002 / 0.132
$\Delta\rho_{max}$ / $\Delta\rho_{min}$, e·Å^{-3}	0.748 / -0.552	4.223 / -1.989
